# Urban Road Surface Condition Sensing from Crowd-Sourced Trajectories Based on the Detecting and Clustering Framework

**DOI:** 10.3390/s24134093

**Published:** 2024-06-24

**Authors:** Haiyang Lyu, Qiqi Zhong, Yu Huang, Jianchun Hua, Donglai Jiao

**Affiliations:** Department of Geographic Information Science, School of Internet of Things, Nanjing University of Posts and Telecommunications, Nanjing 210023, China; hlyu@njupt.edu.cn (H.L.); 1022173217@njupt.edu.cn (Q.Z.); 1023172920@njupt.edu.cn (Y.H.); b21080612@njupt.edu.cn (J.H.)

**Keywords:** road surface condition, crowd-sourced trajectories, urban transportation planning, road maintenance

## Abstract

Roads play a crucial role in urban transportation by facilitating the movement of materials within a city. The condition of road surfaces, such as damage and road facilities, directly affects traffic flow and influences decisions related to urban transportation maintenance and planning. To gather this information, we propose the Detecting and Clustering Framework for sensing road surface conditions based on crowd-sourced trajectories, utilizing various sensors (GPS, orientation sensors, and accelerometers) found in smartphones. Initially, smartphones are placed randomly during users’ travels on the road to record the road surface conditions. Then, spatial transformations are applied to the accelerometer data based on attitude readings, and heading angles are computed to store movement information. Next, the feature encoding process operates on spatially adjusted accelerations using the wavelet scattering transformation. The resulting encoding results are then input into the designed LSTM neural network to extract bump features of the road surface (BFRSs). Finally, the BFRSs are represented and integrated using the proposed two-stage clustering method, considering distances and directions. Additionally, this procedure is also applied to crowd-sourced trajectories, and the road surface condition is computed and visualized on a map. Moreover, this method can provide valuable insights for urban road maintenance and planning, with significant practical applications.

## 1. Introduction

Urban planning aims to create orderly, functional, and sustainable development within urban areas, including efficient transportation that improves mobility and ensures safety for all road users [1,2]. In cities, roads serve as crucial infrastructure, acting as the physical network for the movement of people and cargo [3,4]. The movement of vehicles across the road network results in damage (for example, potholes and distresses) to the road surface [5]. Additionally, various municipal facilities such as manholes and speed breakers are distributed alongside roads [6,7]. All of these elements contribute to different types of bump features of road surface (BFRSs). Information regarding these road features is vital for urban road maintenance and planning.

Therefore, sensing the surface condition to gather information like BFRSs is essential for urban transportation [8,9]. Traditional methods of road surface sensing rely on professional equipment and personnel, such as LiDAR, photogrammetry, and remote sensing [8,10,11,12,13]. These methods are time-consuming and labor-intensive, making it challenging to keep up with the rapidly changing urban road conditions. However, individuals who use these roads also record their actual conditions. These user-generated, crowd-sourced trajectories have become a vital data source for gathering road information [14,15]. Consequently, in recent years, there has been a shift from professional to crowd-sourced equipment for road condition sensing [16]. The use of smartphones as a source of crowd-sourced data for road information is gaining attention [17,18,19,20]. The advantage of smartphones as a source of crowd-sourced data lies in their ability to provide abundant road surface information [21]. However, there are challenges in extracting road features, especially in terms of expressing road surface information and modeling movement information [22,23]. These challenges include effectively combining multiple sensors (GPS, orientation sensors, and accelerometers) of smartphones’ data and detecting and integrating this data [6,24,25,26]. Addressing these three key issues—multi-sensor data fusion, feature detection, and data integration—is crucial within the framework of crowd-sourced data for collecting BFRS and sensing the condition of urban road surfaces.

To address these issues, this article proposes the Detecting and Clustering Framework (DCF) for sensing urban road surface conditions. The method involves collecting BFRSs (Bump Feature Representation Scores) from crowd-sourced trajectories acquired from smartphones. The movement features are then extracted from the recorded trajectories and represented using wavelet scattering transformation [27]. The BFRSs are detected using Long Short-Term Memory (LSTM) networks. Finally, the BFRSs from crowd-sourced trajectories are represented and integrated based on the Density-Based Spatial Clustering of Applications with Noise (DBSCAN). The contributions of this study are as follows: (1) the study explores movement features from recorded trajectories and applies spatial transformation based on the physical structure of multiple sensors; (2) the bump feature is encoded using wavelet scattering transformation, and a neural network is designed to detect BFRSs from crowd-sourced trajectories, with comparisons made to other methods; and (3) the BFRSs from crowd-sourced datasets are represented and integrated with movement information using the proposed two-stage clustering method.

The rest of this article is organized as follows. The subsequent section presents an overview of works related to this article. Section 3 provides a detailed description of the BFRS collection method, which is based on crowd-sourced multi-sensor stream data from smartphones. Experiments and results are discussed in Section 4, followed by conclusions and future work in Section 5.

## 2. Related Work

From the perspective of road information sensing and the related dataset, these methods can be broadly categorized into two dimensions: those oriented toward linear road connectivity (*xy* dimension) and those targeting road surface vertical dimensions (*z* dimension).

(1) Methods focused on linear road connectivity are employed in this study. These methods encompass geometric structure detection [11,28], map construction [18], map matching of trajectories [25,29], connection relations and dead-reckoning [19,20,22], road traffic flow prediction [23,30,31], and a focus on aspects such as the geometric shape of the road network, the topological relation of road junctions, and traffic congestion. Regarding the geometric shape of the road network, computations are usually performed for the centerline of road segments, geometric shape of road connections, and the xy dimensions of curbs. Traditional data collection techniques in this field include field survey measurements, vehicle-borne LiDAR, photogrammetry, and satellite image analysis. Hu et al. [32] extracted the road centerline using LiDAR point cloud by employing a combination of mean shift, tensor voting, and Hough transform methods, while Wei et al. [11] used a CNN-based segmentation framework to segment and convert roads from remote sensing into intensity maps, and extracted road segments through an iterative search strategy. On the other hand, crowd-sourced trajectory data mining methods, using non-professional devices such as smartphones, offer a rapid and convenient alternative [1]. Additional methods and road information can be found in Siegemund et al. [28], Lyu et al. [18], and Wang & Bao et al. [4]. With the help of trajectories, these approaches efficiently extract time-sensitive information such as road network structure, real-time traffic flow, road node connectivity, and vehicle movement routes. Therefore, trajectory-based road information sensing methods are widely used in the aspect of the topological relation of road junctions. Huang et al. [22] computed turn information from trajectories, concentrated trajectory points around intersections, and detected intersections and road segments based on this information. This method deals with the 3D structure of roads and reconstructs the complex road network from the sparsely sampled trajectory. Moreover, crowd-sourced trajectories can be applied to detect current traffic rules at road intersections [19] and enhance the road network with updated road information by identifying existing errors [20]. In such situations, map matching between trajectories and road networks is usually conducted, involving data preprocessing, semantic rule enhancement, and similarity metric computation [29]. However, robust map matching results become difficult to acquire due to signal loss and instability in urban areas, requiring the application of multiple sensors such as inertial measurement units (IMU) [25]. In terms of traffic congestion, linear road connectivity information is crucial. Byun et al. [23] used unmanned aerial vehicle (UAV) images to estimate vehicle speeds based on deep learning neural networks, detecting and tracking vehicle movements by computing the image scale from lane distances. This method is employed for road traffic monitoring. Furthermore, road traffic information can also be computed based on trajectories. Sun et al. [30] matched collected trajectory data to the road network using hidden Markov models (HMMs), computed the average speed of each related road segment, and predicted road traffic congestion using different neural network models, such as Convolutional Neural Networks (CNN), Recurrent Neural Networks (RNN), Long Short-Term Memory (LSTM), and Gated Recurrent Units (GRU). Similar work has also been conducted by Bogaerts et al. [31], who designed and applied a CNN–LSTM neural network for short- and long-term traffic forecasting. This category emphasizes information on road passage dimensions.

(2) Methods targeting road surface vertical dimensions. This category includes methodologies focused on road surface materials, damage conditions, width, and municipal infrastructure information, and professional equipment used includes field measuring devices, LiDAR, and stereovision cameras, along with customized IoT sensor devices [5,6,7,8,10,33,34]. Kuduev et al. [10] and Bhatt et al. [6] utilized LiDAR to collect point cloud data for the road surface, and detected road surface damages based on the threshold value of the distance difference between the local fitted plane and the point cloud surface. Tan et al. [8] collected the road surface information from UAV oblique images, generated the point cloud of road surface based on the photogrammetry theory, and computed the road surface condition using the constructed 3D road surface model. While these methods yield highly accurate road information with precise geometric fidelity, they are limited by the need for specialized equipment and personnel, and are time-consuming and labor-intensive, thus constraining their application in frequent road information updates [2,3,6]. Another kind of road surface information collection method belongs to the image-based methods [7]. Ren et al. [5] computed the road surface damage information from street-view images, based on the improved YOLOv5 network model that uses the generalized feature pyramid network (Generalized-FPN) structure. Such kinds of data acquirement methods improve the efficiency of traditional work. In addition, other kinds of IoT sensors can also be applied to achieve the goal. Mednis et al. [33] designed the RoadMic based on the sound sensor, and collected vibrations of the vehicle during the movement, which can be used to detect potholes and damage of road surfaces. With the continuous development of smartphone hardware capabilities, acquiring road surface information using multi-sensor smartphones has become increasingly feasible [22]. Zang et al. [17] mounted the smartphone on a bicycle, collected road surface information based on the recorded acceleration changes, and derived the road surface changes using the threshold value method. In addition to the location information from smartphone sensors, these methods often require data from supplementary sensors like cameras, accelerometers, gyroscopes, and audio sensors [9,14,26]. Li et al. [16] proposed a road surface detection method based on the continuous wavelet transform (CWT). The collected accelerations were preprocessed with reorientation and noise filtering to acquire actual changes in road surface, then the CWT was applied to the acceleration and the threshold value was used to detect abnormal changes related to the road surface. However, the use of smartphones, a type of non-professional measuring device, introduces considerable noise and lower information density, making the extraction of road surface features and the integration of multi-source data challenging [12,20,25,34]. Since the neural network mode has the capability of feature encoding and detecting, these methods are widely used to detect complex features. Varona et al. [22] designed an experiment to detect potholes in the road surface, and analyzed and applied different deep learning models, including CNN, LSTM, and reservoir computing models, to detect road surface information. Chen et al. [26] detected the road surface information based on the combination of short-time fast Fourier transform and wavelet transform with the CNN, and compared of the results between a professional sensor and the smartphone sensor, which showed the feasibility of the smartphone sensors. Based on the feature encoding and detection capability of the neural network, road information can be collected and represented with the help of crowd-sourced trajectories. 

(3) Methods targeting road surface dimensions are categorized into those focused on road surface materials, damage conditions, width, and municipal infrastructure information. Professional equipment such as field measuring devices, LiDAR, stereovision cameras, and customized IoT sensor devices are used for these methods [5,6,7,8,10,32,33]. Kuduev et al. [10] and Bhatt et al. [6] utilized LiDAR to collect point cloud data for the road surface and detected damages based on the threshold value of the distance difference between a local fitted plane and the point cloud surface. Tan et al. [8] collected road surface information from UAV oblique images, generated a point cloud of the road surface using photogrammetry theory, and computed the road surface condition using a constructed 3D road surface model. While these methods provide highly accurate road information with precise geometric fidelity, they are limited by the need for specialized equipment and personnel, and are time-consuming and labor-intensive, restricting their application in frequent road information updates [2,3,6]. Another category of road surface information collection methods belongs to image-based methods [7]. Ren et al. [5] computed road surface damage information from street-view images using the improved YOLOv5 network model with the Generalized-FPN structure. These types of data acquisition methods improve the efficiency of traditional work. Additionally, other IoT sensors can be applied to achieve the same goal. Mednis et al. [33] designed the Road-Mic, which uses sound sensors to collect vibrations of vehicles during movement. These vibrations can be used to detect potholes and damage on road surfaces. With the continuous development of smartphone hardware capabilities, it has become increasingly feasible to acquire road surface information using multi-sensor smartphones [22]. Zang et al. [17] mounted smartphones on bicycles to collect road surface information based on recorded acceleration changes and derived road surface changes using the threshold value method. These methods often require data from supplementary sensors like cameras, accelerometers, gyroscopes, and audio sensors in addition to location information from smartphone sensors [9,12,26]. Li et al. [16] proposed a road surface detection method based on the continuous wavelet transform (CWT). They preprocessed collected accelerations with reorientation and noise filtering to acquire actual changes in the road surface. Then, the CWT was applied to the accelerations, and a threshold value was used to detect abnormal changes related to the road surface. However, the use of smartphones, which are non-professional measuring devices, introduces considerable noise and lower information density, making the extraction of road surface features and the integration of multi-source data challenging [12,21,26,35]. Neural network models have the capability of feature encoding and detection, making them widely used to detect complex features. Varona et al. [21] designed an experiment to detect potholes on the road surface and applied different deep learning models, including CNN, LSTM, and reservoir computing models, to analyze the road surface information. Chen et al. [26] detected road surface information by combining short-time fast Fourier transform, wavelet transform, and CNN, and compared the results between professional and smartphone sensors, showing the feasibility of using smartphone sensors. With the feature encoding and detection capability of neural networks, road information can be collected and represented with the help of crowd-sourced trajectories.

To obtain information about the condition of the road surface using crowd-sourced trajectories, the road surface information is calculated by detecting, representing, and integrating BFRSs. The trajectory data from multiple smartphone sensors are aligned with the real world and processed with movement information. Then, a feature map is encoded using wavelet scattering transformation and sent to the neural network. Finally, the road surface condition is visualized by representing and integrating the BFRSs from crowd-sourced trajectories. However, it is important to note that crowd-sourced smartphone data are limited in terms of accuracy and quality, and cannot replace professional surveying tools. Therefore, this article will not address the specific shape and measurement accuracy of BFRSs.

## 3. Methodology

### 3.1. Motivation and Background

**Figure 1:**
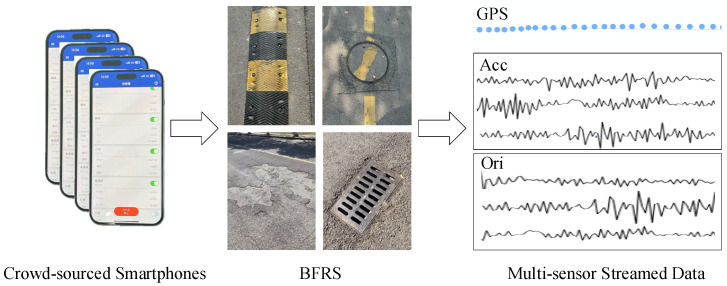
Crowd-sourced multi-sensor trajectories and BFRS.

Suppose the road surface in the local region can be considered as a flat plane, with various features such as surface damage, potholes, and municipal facilities like speed breakers and gutters. These features play a crucial role in urban road maintenance and planning. In this article, BFRSs refer to surface features that have variations in elevation greater than 3 cm and extend horizontally along the road for more than 4 cm. Furthermore, when vehicles pass over these features, the resulting vibrations are recorded by smartphone sensors and form crowd-sourced multi-sensor trajectories, as shown in Figure 1.

**Figure 2:**
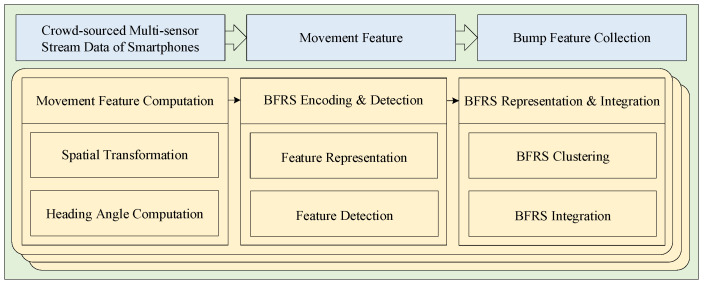
The urban road surface condition sensing workflow based on the DCF.

In these crowd-sourced trajectories, positional and vibrational data are captured using GPS sensors and an IMU sensor that includes accelerometers, magnetometers, and gyroscopes. This information is then used to extract BFRSs and determine their locations. However, extracting BFRSs from the data of multiple sensors on a single smartphone may result in missing information. Nevertheless, by utilizing crowd-sourced trajectories from numerous smartphones, it becomes possible to capture comprehensive BFRSs. Based on the above description, a road surface condition sensing method is proposed, which relies on crowd-sourced trajectories from smartphones. The process begins with a spatial transformation of the accelerations based on orientation data in order to obtain acceleration information perpendicular to the road surface. Then, by analyzing the motion trajectory, the vehicle’s heading direction and motion characteristics are determined. Next, the wavelet scattering transform method is used to extract BFRSs from the acceleration data, which are then input into an LSTM network to link BFRSs with their locations. Finally, a two-stage clustering method is applied to represent and integrate the fusion of this information, and crowd-sourced data are used to extract road surface conditions, ultimately providing the BFRSs and their location information. The workflow of urban road surface condition sensing based on the DCF is illustrated in Figure 2.

### 3.2. Movement Feature Computation for Multiple SensorRecordings

To calculate BFRS from the acquired multi-sensor trajectories, several preprocessing steps are required to prepare the data for analysis. These steps include spatial transformation and heading angle computation. The transformation ensures that the *z*-axis of the accelerometer is aligned perpendicular to the road surface, which is crucial for accurately measuring vertical accelerations that indicate BFRSs. Furthermore, the recorded data from the location sensor is used to determine the vehicle’s heading angle. The specific procedures for carrying out these preprocessing steps are as follows:

(1) Spatial transformation based on the physical multi-sensor structure

**Figure 3:**
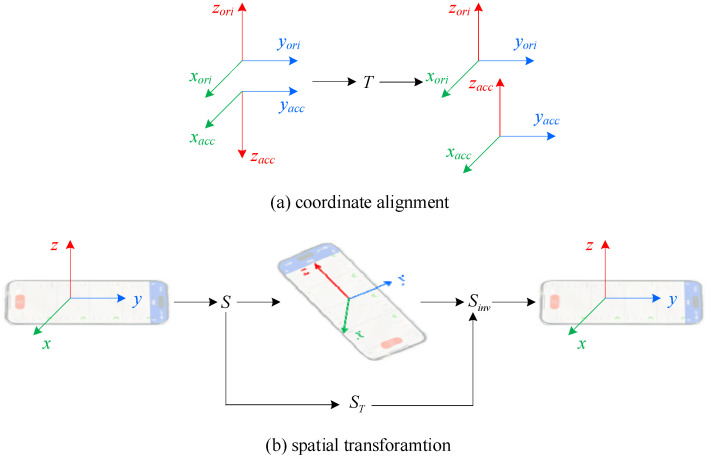
The coordinate alignment and spatial transformation.

In smartphones, the vehicle experiences acceleration changes in both the horizontal direction and perpendicular to the road surface during motion. However, the initial direction of the 3-axis orientation sensor is different from that of the accelerometer, and the directions of the *z*-axis are usually opposite to each other. Additionally, smartphones are randomly placed in vehicles, and all of these factors make it difficult to accurately capture the acceleration changes of BFRSs. Therefore, coordinate alignment and spatial transformation are required to address such situations, as shown in Figure 3.

In Figure 3a, the 3D-axis of orientation is depicted by the right-hand rules coordinate system, with upward direction as the default *z*-axis direction, However, as observed from the recording of acceleration, the default *z*-direction is downward with the same direction of gravity; therefore, coordinate alignment between orientation and acceleration is needed to acquire the actual acceleration changes. Suppose the 3D-acceleration is denoted as *Acc* = [*Accx*, *Accy*, *Accz*], and the process of coordinate alignment can be denoted as Equation (1), where *T* is the 3 × 3 identity matrix with the third diagonal element as −1.

(1) Acc=T×Acc

To eliminate the impact of the arbitrary positioning of smartphones and capture the impact of BFRSs on vehicle dynamics, the acceleration needs to be converted from the sensor coordinate system to the world coordinate system, as shown in Figure 3b. Assuming the smartphone is horizontally placed in the vehicle, with the accelerometer aligned with the world coordinate system, when it is placed in a different orientation, this is recorded by the sensor as *Ori* = $\left[\alpha ,\;\beta ,\gamma \right]$. During the spatial transformation process, the world coordinate system can be determined by the 3D-axis orientation parameters. These parameters enable the construction of a spatial extrinsic rotation matrix *S*, which transforms the acceleration of any spatial orientation to the current coordinate system, in the order of *ZYX*, as denoted in Equation (2).

(2) $$S=\left[\begin{array}{ccc} cos\left(\alpha \right)cos\left(\beta \right) & cos\left(\alpha \right)sin\left(\beta \right)sin\left(\gamma \right)-sin\left(\alpha \right)cos\left(\gamma \right) & cos\left(\alpha \right)sin\left(\beta \right)cos\left(\gamma \right)+sin\left(\alpha \right)sin\left(\gamma \right)\\ sin\left(\alpha \right)cos\left(\beta \right) & sin\left(\alpha \right)sin\left(\beta \right)sin\left(\gamma \right)+cos\left(\alpha \right)cos\left(\gamma \right) & sin\left(\alpha \right)sin\left(\beta \right)cos\left(\gamma \right)-cos\left(\alpha \right)sin\left(\gamma \right)\\ -sin\left(\beta \right) & cos\left(\beta \right)sin\left(\gamma \right) & cos\left(\beta \right)cos\left(\gamma \right) \end{array}\right]$$

In other words, the recorded acceleration data are the spatial transformed data, and they need to be transformed back to the world coordinate system, with the inverse matrix *S_inv_*. However, the 3D axis of the orientation sensor are vertical to each other, i.e., the rotation matrix *S* forms an orthogonal matrix. Hence, the inverse matrix *S_inv_* can be calculated simply by the transpose of the rotation matrix, i.e., *S_inv_* = *S_T_*, and the spatial transformation process for the acceleration from an arbitrary coordinate system to the world coordinate system can be performed with the following equation.

(3) $$Acc=S_{T}*Acc$$

(2) Heading angle computation based on the spherical trigonometry

In the context of bi-directional travel, BFRSs can be found on roads that have different travel directions. To account for this, the heading angle is calculated based on the vehicle’s travel direction in relation to the north direction on the horizontal plane. The heading angle is measured as the clockwise angle from north. Since GPS locations are recorded in longitude and latitude, the heading angle *θ* between points *p*_1_(*lat*_1_, *lon*_1_) and *p*_2_(*lat*_2_, *lon*_2_) can be computed based on spherical trigonometry [36]. 

(1) Convert locations from degrees to radians based on *radians* = *degrees* × π/180, and represent the coordinate as *p*_1_($\phi_{1}$, $\lambda_{1}$) and *p*_2_($\phi_{2}$, $\lambda_{2}$).

(2) Apply the spherical trigonometry formula to calculate the heading angle *θ* based on Equation (4):

(4) $$\theta =\arctan\left(\sin\left(\Delta \lambda \right)\cdot \cos\left(\phi_{2}\right),\cos\left(\phi_{1}\right)\cdot \sin\left(\phi_{2}\right)-\sin\left(\phi_{1}\right)\cdot \cos\left(\phi_{2}\right)\cdot \cos\left(\Delta \lambda \right)\right)$$

where Δ*λ* = *λ*_2_ − *λ*_1_, and arctan is an arctangent function that can handle angles in all four quadrants, with a return value between −π and π. 

(3) Convert calculated radians back to degrees based on *degrees* = *radians* × 180/π.

(4) Adjust the angle to a range between 0 to 360 degrees, and this can be performed by checking the value of *θ* and adding 360 degrees accordingly, as denoted by Equation (5).

(5) θ = mod(θ+360, 360)

where mod function returns the remainder after the division operation, and meets the demand of θ∈[0,360].

With the heading angle information, BFRSs distributed in opposite directions of the pathway can be restored, which will be described in the following sections.

### 3.3. BFRS Feature Encoding and Detection Based on Neural Network

After transforming the multi-sensor trajectories spatially, it becomes necessary to choose the acceleration for detecting BFRSs. However, it is crucial to consider the instantaneous changes in acceleration for extracting BFRSs, as the acceleration data may contain noise and their quality can be inconsistent during sampling. Applying direct filtering to remove the noise might unintentionally eliminate some of the useful BFRSs. Therefore, it is essential to preprocess the acceleration data before inputting it into the neural network. The specific steps for this are as follows:

(1) Feature representation based on the wavelet scattering transformation

To encode the acceleration features, it selects the wavelet scattering transformation, and extracts the wavelet scattering spectrum matrix as the feature encoding result for the bump feature. The method combines the multi-scale analysis properties of wavelet transforms with the hierarchical structure of deep learning, and the basic principles of the wavelet scattering transform include the following: (1) wavelet transform, which provides localized information in both time and frequency domains, and uses a series of “wavelet” functions at different scales instead of the learnable convolution kernels of CNN and (2) hierarchical structure, and each layer of the network applies a set of wavelet filters, then computes the modulus of each filter’s output, which extracts features through a deep network structure without involving training. The transformation process is as follows:

(1) Pass the acceleration through a set of wavelet filters that localize the data *x* at different scales, and represent the bump feature as a series of wavelet coefficients that capture local features of the input data, as denoted by Equation (6), where $\psi_{j}\left(t\right)$ is the wavelet function, and *j* represents the scale. Then, apply a modulus operation to each wavelet coefficient (denoted as $U1[j]x\left(t\right)$) and maintain the transformation’s stability to local changes in the input data.

(6) $$U1[j]x\left(t\right)=\left|x*\psi_{j}\left(t\right)\right|$$

(2) Process these modulus values through a low-pass filter $\phi \left(t\right)$ to extract smooth features, and obtain the result $S1x\left(t\right)$, as denoted by Equation (7).

(7) $$S1x\left(t\right)=U1[j]x\left(t\right)*\phi \left(t\right)$$

(3) Apply a similar process of second and higher scattering layers to the output of the first layer but after the modulus operation, and obtain the result $U2[j,k]x\left(t\right)$, which allows the capture of more complex and advanced features of the data, as denoted by Equation (8). Then, repeat the low-pass filtering again, and obtain the result $S2x\left(t\right)$, as denoted by Equation (9).

(8) $$U2[j,k]x\left(t\right)=\left|U1[j]x\left(t\right)*\psi_{k}\left(t\right)\right|$$

(9) $$S2x\left(t\right)=U2[j,k]x\left(t\right)*\phi \left(t\right)$$

**Figure 4:**
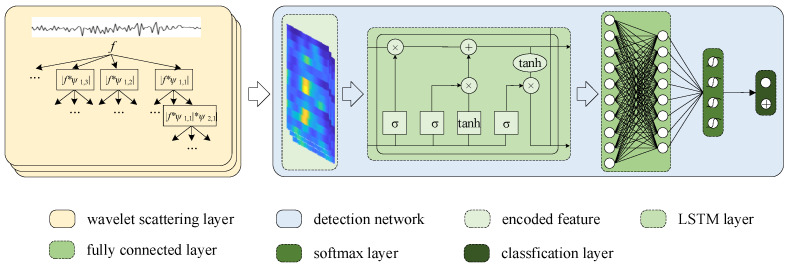
The network architecture for BFRS detection.

The wavelet scattering transform extracts complex patterns of acceleration in a hierarchical manner, ensuring stability to input variations without the need for the training process commonly used in deep learning models. Therefore, this article employs the wavelet scattering transform to encode features of changes in acceleration, as demonstrated in Figure 4.

(2) BFRS detection from trajectories based on the LSTM neural network

The encoded feature is then input into the LSTM neural network to extract features from trajectories with temporal characteristics, allowing for the identification of BFRSs. LSTM is a specialized type of RNN that is well-suited for handling and predicting long-term dependencies in sequence data. Within the network, LSTM operates on the principle of a “memory cell” which can retain its state over extended periods of time. Each memory cell comprises multiple components, including one or more “gate” structures (such as the input gate, forget gate, and output gate) as well as a cell state. The BFRS detection process, based on LSTM, involves the following steps:

(1) First, decide which information to discard from the cell state based on the forget gate. It makes this decision based on the current input *x_t_* and previous output *h_t−_*_1_, as denoted by Equation (10), where σ is the sigmoid function and *W_f_* and *b_f_* are the weights and biases of the forget gate.

(10) $$f_{t}=\sigma (W_{f}\cdot \left[h_{t-1},x_{t}\right]+b_{f})$$

(2) Next, decide which new information is stored in the cell state based on the input gate, and update the value by the sigmoid layer. Then, create a new candidate values vector ${\tilde{C}}_{t}$ by the tanh layer, as denoted by Equation (11).

(11) $$i_{t}=\sigma (W_{i}\cdot \left[h_{t-1},x_{t}\right]+b_{i}),{\tilde{C}}_{t}=tanh(W_{C}\cdot \left[h_{t-1},x_{t}\right]+b_{C})$$

(3) Update the old cell state $C_{t-1}$ to the new cell state $C_{t}$. The old state is multiplied by $f_{t}$ to “forget” certain information, and then new candidate values are added by multiplying $i_{t}$ and ${\tilde{C}}_{t}$, as denoted by Equation (12).

(12) $$C_{t}=f_{t}*C_{t-1}+i_{t}*{\tilde{C}}_{t}$$

(4) Finally, determine which part of the cell state will be included in the output. The output is determined by the cell state, but it will first pass through the sigmoid layer to select the relevant part, as indicated by Equation (13).

(13) $$o_{t}=\sigma (W_{o}\cdot \left[h_{t-1},x_{t}\right]+b_{o}),h_{t}=o_{t}*tanh(C_{t})$$

In these steps, *W* and *b* represent weights and biases, and “*” denotes element-wise multiplication. Due to its excellent ability to handle long sequence data, LSTM is widely used in areas such as time series prediction. Therefore, this article utilizes an LSTM neural network to detect BFRSs, as illustrated in Figure 4.

In Figure 4, there are two main steps in the network architecture: 

(1) Encoding the features. The acceleration data are inputted into the wavelet scattering transform model, which automatically encodes the features using multiple wavelet functions at different scales. To calculate the local feature encoding for each consecutive recording, a sliding window with a width of 170 is used, an invariance scale of 0.5 is set, and an oversampling factor of 2 is applied to the recorded acceleration of 100 Hz.

(2) Detecting the features. The encoded features are then passed to the LSTM. The hidden LSTM layer is set to 512, followed by a fully connected layer, a softmax layer, and a classification layer. Finally, each recorded position is assigned a probability of being a BFRS. The results are stored in a buffer queue with a length of 10, and the current result is considered a true BFRS if the number of detections exceeds the threshold (e.g., 5).

### 3.4. BFRS Representation and Integration Using Two-Stage Clustering

With the initially detected BFRSs of Section 3.3, interpreting the road surface condition is still difficult due to the quality-unstable recordings and the need for further representation and integration of the specific location of BFRSs. Additionally, the BFRS is derived from a single data source, which has limited coverage of the road surface. Therefore, to obtain comprehensive BFRS information, the crowd-sourced data are processed using the proposed neural network model, resulting in the detection of multiple, inconsistent BFRSs. To address these issues, the two-stage clustering method DBSCAN is proposed. DBSCAN is a density-based spatial clustering algorithm that can identify regions of high density as clusters and consider low-density areas as outliers or noise. There are two main concepts in the algorithm: (1) a core point *p* that is considered a core point of dataset *D* if it has a high enough number $\left|N_{\varepsilon }\left(p\right)\right|\;$of points *q* within its neighborhood $N_{\varepsilon }\left(p\right)$ of distance *ε*, as denoted by Equation (14), and (2) it is density-reachable, in that the relationship of a point *q* can be reached from a core point *p* by a chain of points {$p_{1}$, $p_{2}$, …, $p_{n}$}$\in N_{\varepsilon }\left(p\right)$ with sufficient density condition *C*, as denoted by Equation (15). The specific process of DBSCAN is as follows:

(1) First, determine two parameters: the neighborhood radius *ε* and the minimum number of points $N_{min}$.

(2) For each point *q* in the dataset *D*, calculate the number of points within its neighborhood distance *ε*. If this count $\left|N_{\varepsilon }\left(p\right)\right|$ is greater or equal to $N_{min}$, mark it as a core point *p*.

(3) For each core point *p*, identify all density-reachable points *q* starting from it to form a cluster. Repeat this process until all core points have been visited.

(4) Among the non-core points *q*, those within the *ε* neighborhood of any core point *p* are marked as border points; others are considered noise points.

(14) $$\left|N_{\varepsilon }\left(p\right)\right|\geq N_{min}\;s.t.\;N_{\varepsilon }\left(p\right)=\{q\in D|dist\left(p,q\right)\leq \varepsilon \}$$

(15) $$C:\;\exists \{p_{1},\;p_{2},\;\ldots ,\;p_{n}\}\;p_{1}\;=\;p,\;p_{n}\;=\;{q\;\Lambda p}_{i+1}\in N_{\varepsilon }\left(p\right)$$

**Figure 5:**
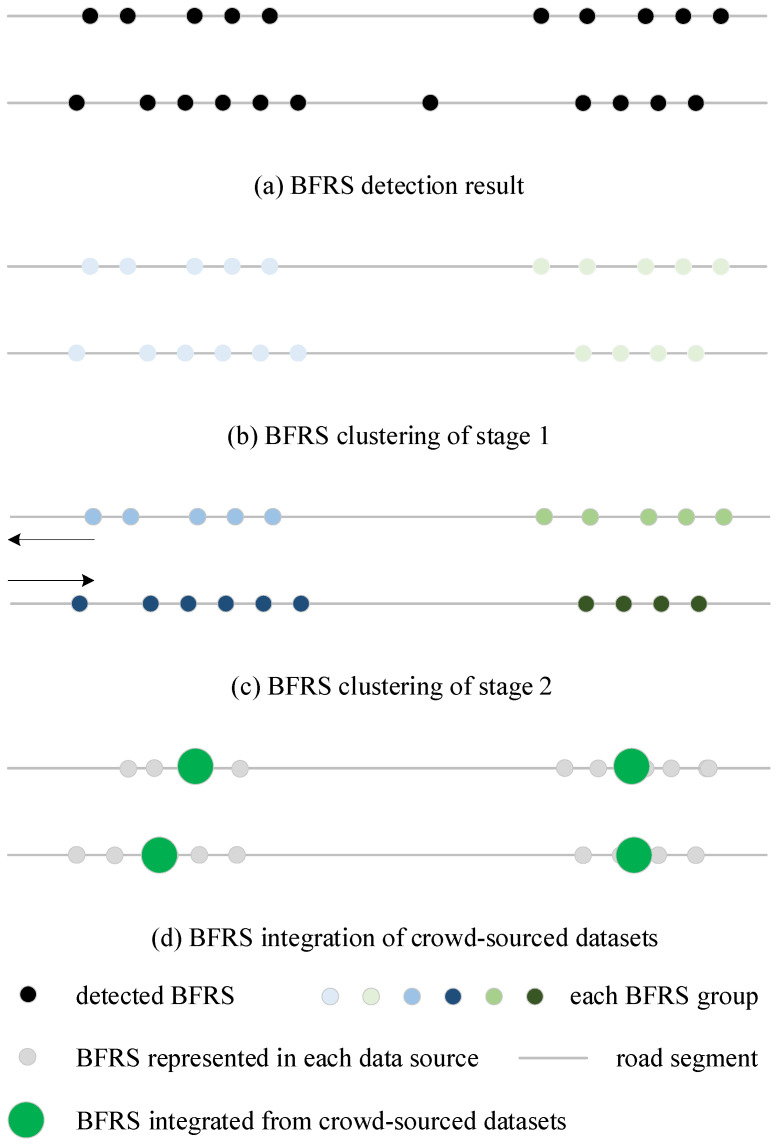
The BFRS clustering process.

Based on the clustering method, the initial detection results are grouped according to distance in the first stage. In the second stage, each clustering group is re-clustered using road traffic direction information and heading angle information. The proposed two-stage clustering method is repeated to handle the crowd-sourced datasets, based on the BFRSs detected from different trajectories, as shown in Figure 5.

As shown in Figure 5, the BFRS representation and integration processes involve two stages of clustering operations. The first clustering fusion operation (Figure 5b) merges the detection results (Figure 5a) into BFRSs, and the second clustering fusion operation (Figure 5c) merges detection results from crowd-sourced data into BFRSs (Figure 5d).

(1) In the single dataset

(1) By applying the clustering method that relies on spatial proximity distance, the sampling points detected by the LSTM network model can be clustered. The clustering distance is usually set to 10 m.

(2) For each cluster result, the clustering method based on the heading angle is used to cluster the result. The clustering angle is typically set to 90°, representing BFRSs on lanes with different travel directions.

After the operation of a single dataset, the detection points of each group are fused using the detection probability as the weight. If the number of clustered points in each group is less than the threshold (e.g., 10), the LSTM detection results are converted to BFRSs.

(2) In the crowd-sourced datasets

(1) This uses the clustering method based on spatial proximity distance to cluster the BFRSs from crowd-sourced data, with the clustering distance typically set to 10 m.

(2) For each cluster result, the clustering method based on the heading angle is used to cluster the results, with the clustering angle typically set to 90°.

After performing the representation and integration operation, the points of each cluster are fused according to the number of points in each DBSCAN cluster. The fused results are then considered as the final detection outcome, as shown in Figure 5.

### 3.5. BFRS Collection Using Non-Homogeneous Spectrum Feature

The main steps of the urban road surface sensing method described in this article can be summarized into three phases: (1) The computation of movement features for multiple sensor recordings. This initial phase involves spatially transforming the raw trajectory data to obtain acceleration aligned with the world coordinate system and the vehicle’s heading angle. (2) The encoding and detection of BFRSs based on a neural network. In this phase, the acceleration undergoes a wavelet scattering transformation to effectively encode the acceleration features. The transformed data are then fed into an LSTM network to detect BFRSs. (3) The representation and integration of BFRSs using a two-stage clustering approach. The final phase focuses on representing and integrating detection results from single data sources and further fusing BFRS results from multiple sources. The specific algorithm is detailed below, as outlined in Algorithm 1:
**Algorithm 1** The urban road surface condition sensing based on the DCF**Input:** crowd-sourced trajectories from smartphone T $\left\{P\left(lon,lat\right),Ori\left(\alpha ,\beta ,\gamma \right),Acc\left({Acc}_{x},{Acc}_{y},{Acc}_{z}\right)\right\}$**Output:** road surface condition represented by BFRS {*BFRS*}**FOR** trajectory(*i*) IN T     //phase1: movement feature computation for multiple sensor recordings     Acc=Allign(Acc); //coordinate alignment based on Equation (1)     Acc=SpatialTransform(Ori,Acc); //spatial transformation based on Equations (2) and (3)     θ=ComputeAngle(P); //heading angle computation based on Equations (4) and (5)     //phase 2: BFRS feature encoding and detection based on neural network     **FOR** recording IN trajectory          *feature* = WaveletScatteringTransform(Acc); //feature encoding based on Equations (6)–(9)          *BFRS_detected_* = LSTM(*feature*); //feature detection based on Equations (10)–(13)     **END**     //phase 3: BFRS representation and integration using two-stage clustering     *BFRS(i)_group_* = Cluster(*BFRS(i)_detected_*, *d*); //fist stage—clustering based on Equations (14) and (15)     *BFRS(i)_cluster_* = Cluster(*BFRS(i)_group_*, *θ*); //second stage—clustering based on Equations (14) and (15)**END**//BFRS representation and integration for crowd-sourced dataset*BFRS_group_* = Cluster(*BFRS(i)_cluster_*, *d*); //fist stage—clustering based on Equations (14) and (15){*BFRS*} = Cluster(*BFRS_group_*, *θ*); //second stage—clustering based on Equations (14) and (15)**RETURN** {*BFRS*}//road surface condition represented by BFRS


## 4. Experiments and Discussion

This section focuses on experiments of crowd-sourced trajectories in the research area based on the DCF. Following the methodology described in this article, the data undergo spatial transformation and the calculation of direction angles as the initial steps. Subsequently, the acceleration is encoded using the wavelet scattering transform method, and the encoded results are then inputted into an LSTM neural network. Finally, the detection results are clustered using the two-stage clustering method, compared with results from other BFRS detection methodologies, and analyzed for performance across multiple data sources.

### 4.1. The Crowd-Sourced Trajectories and the Research Area

**Figure 6:**
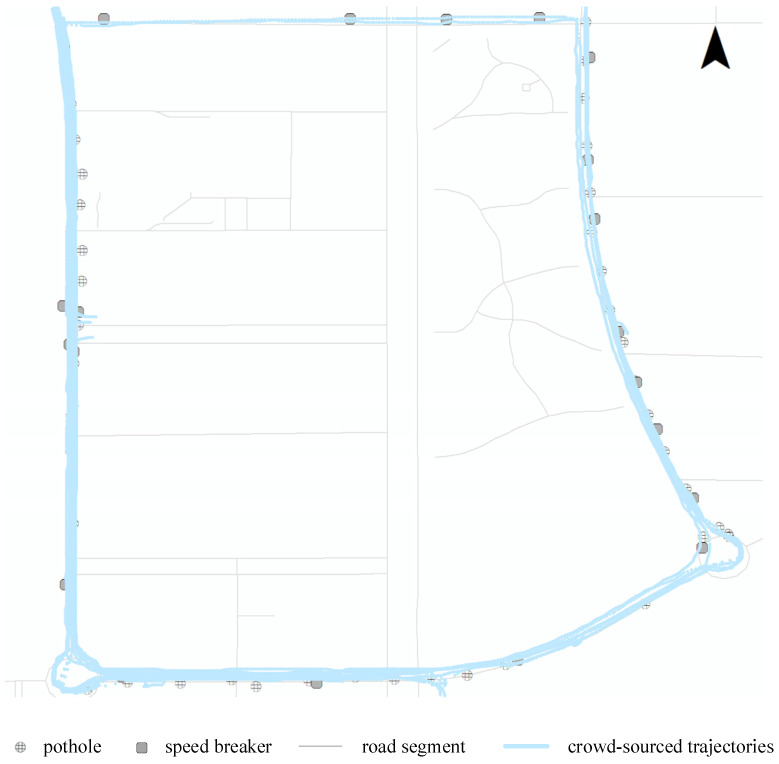
The research area and crowd-sourced trajectories.

The research area and crowd-sourced trajectories are depicted in Figure 6. The original dataset has a sampling rate of 1 Hz for the GPS sensor, while the accelerometers and orientation sensors operate at a sampling frequency of 100 Hz. The recorded results consist of geographic coordinates, 3D-axis acceleration, and 3D-axis orientation angles. To synchronize the data from different sensors, linear interpolation is used on the orientation and location sensor data, leveraging the time stamps from the accelerometer.

In Figure 6, there are 21 speed breakers and 44 potholes distributed along the road in an area *A* of 290,972 m^2^. Four smartphones, each equipped with GPS and IMU sensors, are used to record road surface conditions. Due to the varying characteristics and precision levels of each device, datasets that contain explicit errors—such as significant deviations of GPS locations from their associated road segments—are manually discarded. Finally, 32 valid trajectories (*N_traj_*) of the dataset *Ds* were collected in this area, with a total length *L_traj_* of 33,559 m, which is significantly longer than the road segment length *L_A_* of 2410 m. A total of 3898 GPS recordings (*R_GPS_*) were collected, along with 389,780 recordings of the acceleration (*R_acc_*_(3-axis)_) and the orientation (*R_ori_*_(3-axis)_), as shown in Table 1. Furthermore, the training dataset for the neural network was selected from outside the research area to maintain the independence of the network model.

### 4.2. Spatial Transformation and Heading Angle Computation

**Figure 7:**
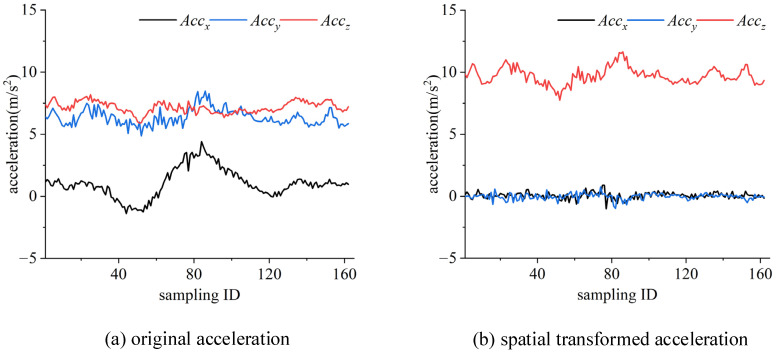
The acceleration data before and after spatial transformation.

To determine the condition of the road surface using crowd-sourced trajectories, we apply spatial transformation to the multi-sensor recordings from smartphones. We also compute the heading angle to restore movement information. Based on the spatial transformation parameters derived from the orientation angles, we construct an inverse matrix for the acceleration transformation. This process allows us to transform the orientation of a smartphone, regardless of its initial position, into a horizontal orientation. Subsequently, we utilize the three-axis acceleration, which is aligned perpendicular to the road surface after the transformation, to detect BFRSs. After undergoing spatial transformation, the mean value of the *z*-axis acceleration becomes approximately 9.8 m/s^2^, closely aligning with the actual acceleration due to gravity. The spatial transformation process is illustrated in Figure 7.

In Figure 7a, it is difficult to judge the actual changes of the road surface as the acceleration in the *x*-axis, *y*-axis, and *z*-axis are changing without any regular pattern due to the arbitrary placement of the smartphone. However, it becomes explicit after the spatial transformation in Figure 7b as the acceleration in the *x*-axis and *y*-axis remain steady while the acceleration in the *z*-axis continues to change. Additionally, it can be clearly observed that there are bump features in the recordings.

**Figure 8:**
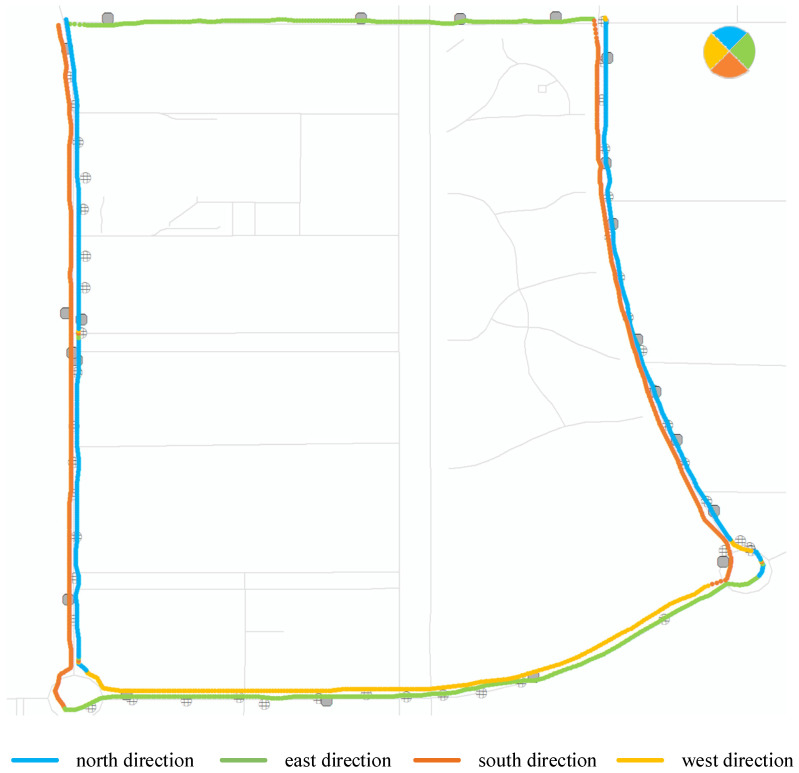
The movement information of heading angle.

On the other hand, according to the principles of spherical trigonometry, the vehicle’s heading angle is calculated using latitude and longitude information. The results for different heading angles are then visualized, based on the magnitude of the angle, as shown in Figure 8.

In Figure 8, the heading angles of different trajectory segments are computed, and four directions, north, east, south, and west, are visually represented using different colors. The result allows for the observation of movement directions based on the heading angle, in accordance with the traffic rule for vehicles to keep to the right. Therefore, the heading angle can be utilized to identify BFRSs that are distributed on different sides of the road.

### 4.3. BFRS Feature Encoding and Detection

**Figure 9:**
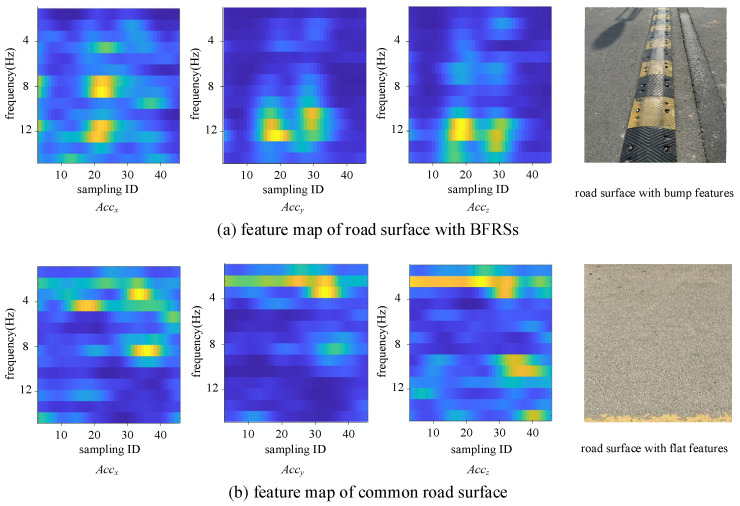
The feature map of different road surfaces based on wavelet scattering transformation. The value in feature map from low to high are represented by the color ranging from blue to yellow.

With the spatially transformed 3D-axis acceleration, the wavelet scattering transformation is applied to encode the data. Following Algorithm 1, a sliding window is used to capture local changes in the recordings, with the following parameters: {window length: 170, invariance scale: 0.5, oversampling factor: 2}. The encoded features are then sent to the detection network, and BFRSs (brake failure-related signals) are detected using the same structure described in Section 3.3. The training options for the LSTM model are set as follows: {initial learning rate: 0.0001, max epoch: 300, mini batch size: 50}. To observe the feature encoding result in detail, two different types of road surfaces are selected and the feature map is visualized in Figure 9. The color ranges from blue to yellow, indicating values from small to high. The higher the value, the yellower the feature map.

As seen from the results, the accelerations of the *x*-axis, *y*-axis, and *z*-axis are processed using the wavelet scattering transformation. The feature maps of the road surface with BFRSs are computed and depicted in Figure 9a, in comparison with those of the road surface with a flat surface in Figure 9b. There are higher values (represented by the color yellow) in the high-frequency domain of the road surface with BFRSs in Figure 9a, while higher values are concentrated in the low-frequency domain. It can be observed that the BFRSs are different from those of the common road surface, with salient high values in the high-frequency domain for the BFRSs compared to the common road surface. In cases of varying driving speeds, acceleration recordings of the same BFRS can differ; for example, recordings at high speeds may appear ‘compressed’ due to shorter sampling times compared with those at low speeds. Although directly detecting BFRSs from acceleration recordings can be challenging, distinct characteristics can emerge in different frequency domains, as represented by the feature map computed from the wavelet scattering transformation. These feature differences make it easy to distinguish the different features of the road surface and contribute to the accurate detection of the neural network.

**Figure 10:**
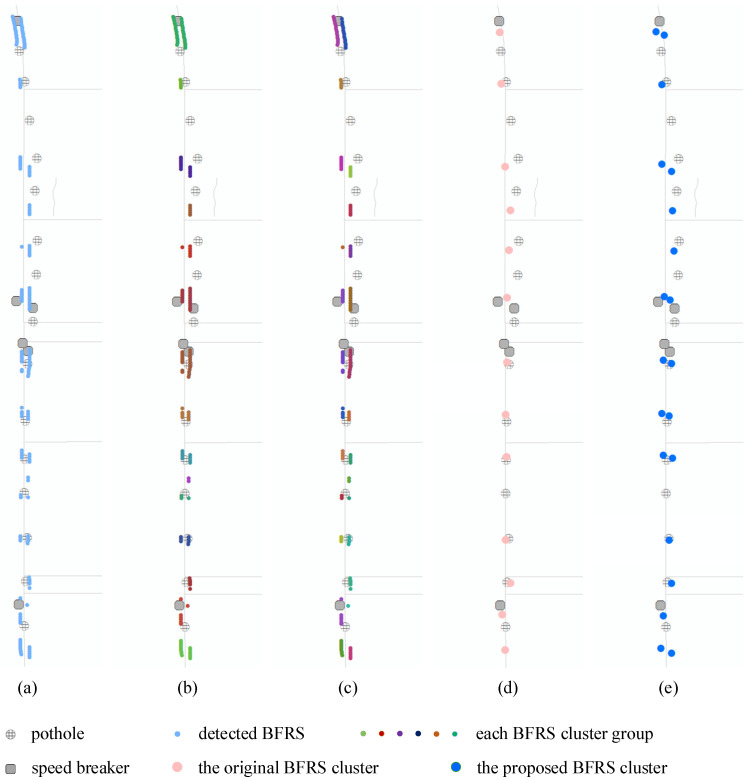
The BFRS representation and integration process. (**a**) the initial detection result, (**b**) the first stage of the clustering process, (**c**) the second stage of the clustering process, (**d**) the clustering result based on the original cluster method, (**e**) the clustering result based on the proposed two-stage cluster method.

Based on the neural network, BFRSs are detected and the initial results are discreetly distributed recordings, as shown in Figure 10. Consequently, the results are further represented and integrated using a two-stage cluster method described in Section 3.4. In Figure 10a, the initial detection result is visualized as a small light blue dot. In the first stage of the clustering process, the results are clustered into groups based on a distance of 10 m, and there is no difference for the BFRSs in the same group, as depicted in Figure 10b. However, this is not the case for BFRSs that are distributed on both sides, such as the speed breaker and the gutter, which are transversely distributed along the road. Furthermore, some road damage may also be consecutively distributed along the road. To address this, the second stage of the clustering process is based on the heading angle information computed in Section 4.2. The parameters of the cluster method are set as {distance: 10 m, angle difference: 90, minimum number of points: 3}, and integration is computed based on the weighted detection probability. The clustering result is depicted in Figure 10c. To compare the difference between the original cluster method and the proposed two-stage cluster method, two clustering results are shown in Figure 10d and Figure 10e, respectively.

In Figure 10d, it shows the clustering result of the original cluster method. The BFRSs in the same group as the result in Figure 10b are simply combined into one position. However, in the result of 10e, only the BFRS group in Figure 10c is combined, indicating differently distributed BFRSs, especially for speed breakers. The same potholes are clustered into two groups, suggesting that the object may be placed in the center of the road surface. From the results, it can be observed that the BFRSs are clustered and differentiated based on the differences in heading angles. BFRSs on different sides of the road are also detected.

### 4.4. Comparison with Different Methods

**Figure 11:**
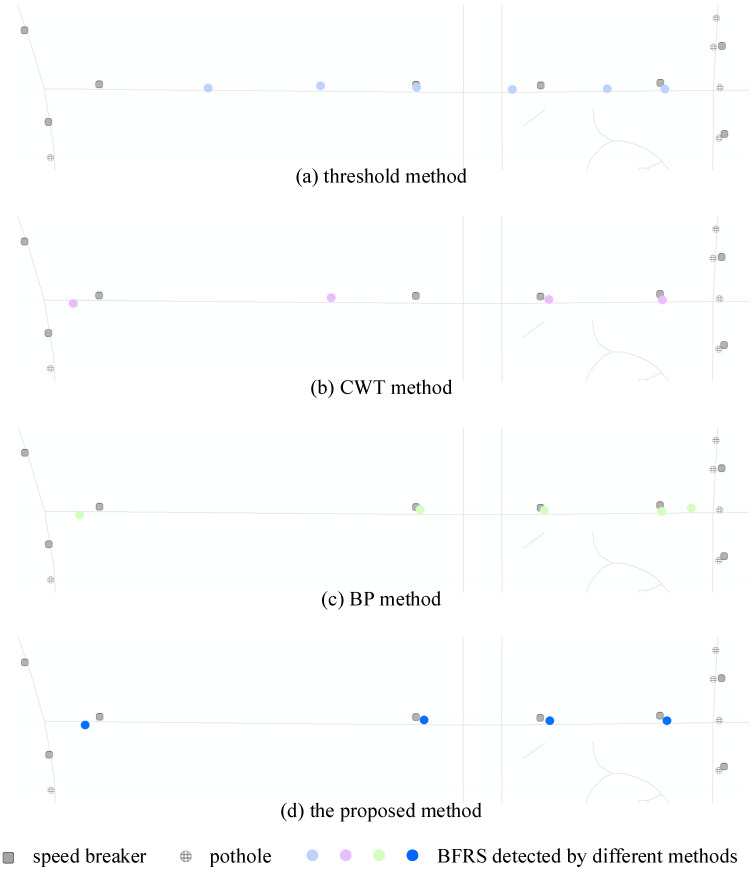
The comparison of different BFRS detection methods.

To evaluate the effectiveness of the proposed method for sensing road conditions, experiments were conducted in an area where four specific BFRSs were present along the road. The results of detecting different BFRSs were compared. In the test area, a ground truth road dataset was used, consisting of four speed breakers that could be accurately recorded by smartphones while traveling. It was expected that these BFRSs would be detected in both driving directions of the road without any confusion if the method performed well. However, it should be noted that the BFRS of potholes may not be completely recorded by different trajectories, as they may only be present on one side of the road. The results are shown in Figure 11.

In Figure 11a, BFRSs are derived using an adaptive threshold value [6], which is calculated based on abnormal changes in acceleration within its surrounding area. The calculation involves taking the first derivative of the acceleration data to identify any abnormal variations, in addition to considering the overall standard deviation of the dataset. Then, the abnormal changes larger than the standard deviation are adaptively selected as BFRSs, and six BFRSs are detected in the result, while only two of them are correct. In Figure 11b, the result is based on CWT [16], with the parameters {wave name: db3, total scale: 256}. The threshold value is adaptively computed based on the absolute mean value of the coefficient matrix, and only the acceleration recording with its related coefficient exceeding ten times the threshold value is chosen as a BFRS. Finally, four BFRSs are detected, and half of them are correct ones. In Figure 11c, the BP neural network [35] is applied to detect BFRSs, with {input dimension: 3, hidden layers: 20}, using the same training dataset as the proposed method. In the result, five BFRSs are detected, and three are considered to be correct BFRSs, while the other two BFRSs seem to be a little far away from the ground truth dataset. The BFRS detection result of the proposed method is illustrated in Figure 11d, with four correct BFRSs detected. It can be observed that the proposed method outperforms other methods in the experiment.

### 4.5. Road Surface Condition Sensing from Crowd-Sourced Datasets

**Figure 12:**
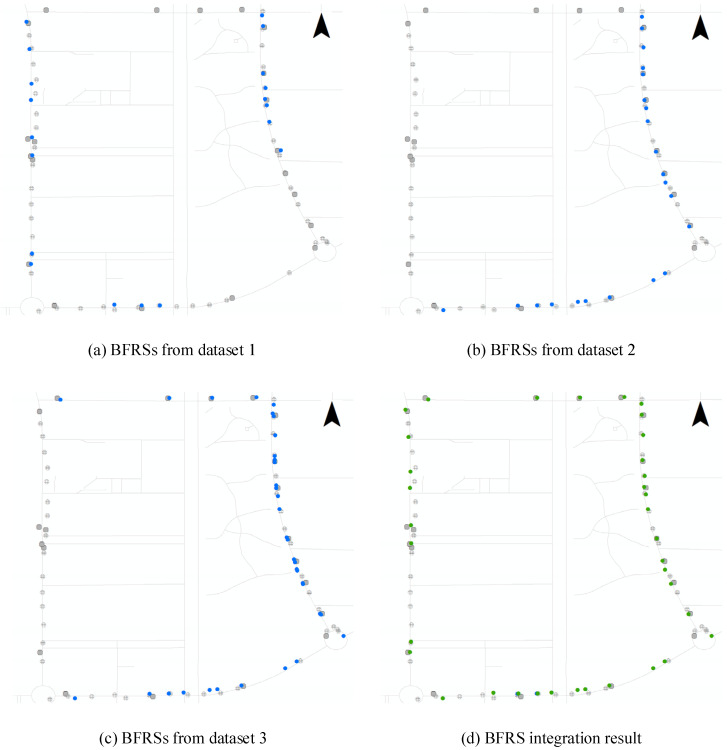
BFRS integration process from different datasets.

Regarding the crowd-sourced data source, the BFRSs detected from a single data source are further integrated based on the two-stage clustering process described in Section 3.4. The BFRSs from the single data source are firstly clustered based on a distance of 20 m with a minimum number of points as 1. Then, the second stage of clustering is applied to each group, with an angle difference of 90 and a minimum number of points as 1. The cluster center integration is computed based on weighted averaging, where the number of detected points in each BFRS is taken as the weight, and the specific locations are computed based on the weight and locations of BFRSs in each group. With the total parameters set as {distance: 20 m, angle difference: 90, minimum number of points: 1}, three different trajectories are processed based on the proposed method and further clustered as the final result, which is depicted in Figure 12.

In Figure 12, we can see three BFRS detection results. The BFRSs are distributed along the road, near the ground truth dataset. In Figure 12a–c, some of the ground truth BFRSs are not fully detected in each trajectory. This is because the original dataset does not provide complete coverage, particularly in the north part of Figure 12a,b and the west part of Figure 12b,c. However, there is a change in Figure 12d, where BFRSs in most of the circle road segments within the research area are detected from multiple data sources. Additionally, there are still some BFRSs that are not detected in the result, even when the road segment is covered by the trajectory, such as the south-east part of Figure 12a. This is a common occurrence during the BFRS detection process, as it is not possible to accurately detect BFRSs with 100% accuracy from single trajectories. Additionally, as crowd-sourced datasets from different devices vary in quality, thresholds are necessary and applied during the clustering process. In the clustering results, any cluster center that falls below the threshold of 3 detected recordings is discarded, since there are few confident detection results. With the help of the crowd-sourced dataset, more BFRSs are correctly detected compared to using a single data source.

**Figure 13:**
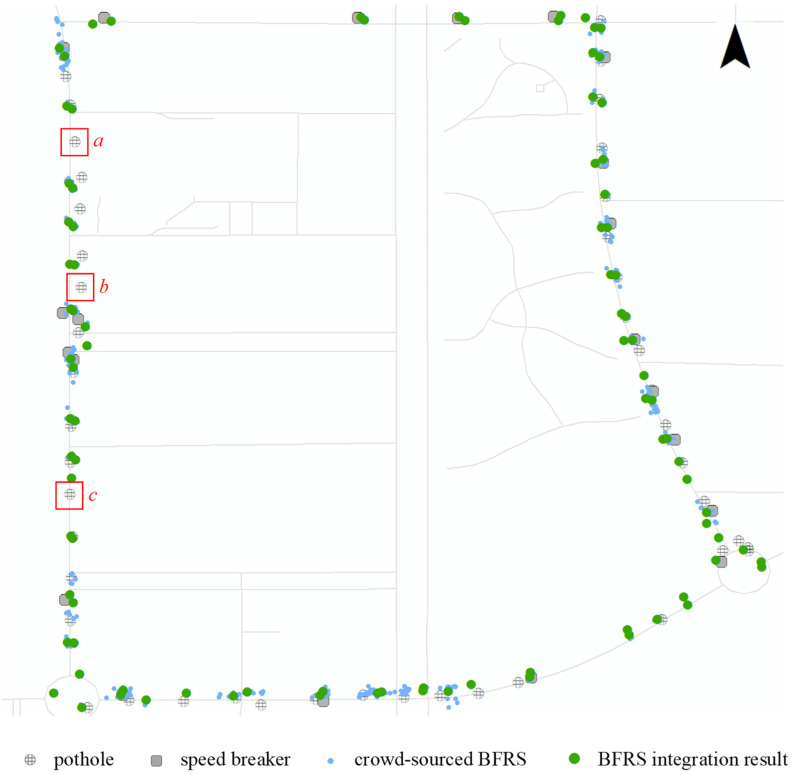
The road surface sensing result from crowd-sourced datasets. The red rectangles of *a*, *b*, *c*, refer to the undetected BFRS.

Based on the proposed method for sensing road surface conditions, the collected 32 trajectories were used to experiment with BFRSs, as shown in Figure 13. It can be observed from the figure that the crowd-sourced BFRSs (small blue dots) are represented and integrated with BFRSs from the research (large green dots), which are consistent with the ground truth dataset. BFRSs that are distributed transversely along the road (such as speed breakers) or objects located near the center of the road (such as potholes) are represented in pairs, while some potholes located on one side of the road are represented by a single green dot. As seen in Figure 12 and Figure 13, not all BFRSs may be detected from a single trajectory, and the detected locations of the same BFRS can vary across different detection results. However, the locations of related BFRSs can be clustered and represented as the cluster center, and the more datasets that are acquired, the higher the confidence in the detection results.

(16) $$Precision=\frac{TP}{TP+FP}$$

(17) $$Recall=\frac{TP}{TP+FN}$$

(18) $$\mathrm{F-score}=2*\frac{Precision*Recall}{Precision+Recall}$$

Detailed statistics of the BFRSs from crowd-sourced trajectories are conducted, and only the BFRS within 10 m of the ground truth dataset are taken as the correct ones. Then, the result is categorized into true positives (TT BFRSs, i.e., the true BFRSs that were correctly detected), false positives (FT BFRSs, i.e., the BFRSs that were falsely detected), and false negatives (TF BFRSs, i.e., the true BFRSs that were not detected). The analysis further evaluates the performance using *Precision* and *Recall* metrics, which are critical for assessing the effectiveness of the detection method, based on Equations (16) and (17). Finally, the *F-score* is computed based on these two quality indexes, as denoted in (18), and the result is illustrated in Table 2.

**Figure 14:**
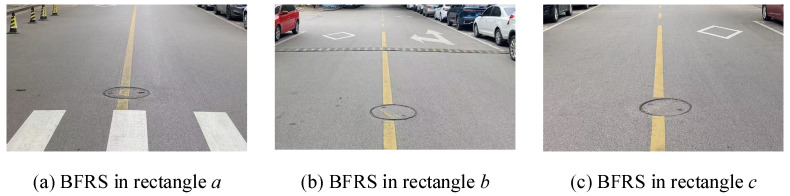
The undetected BFRSs in the research area.

As shown in the table, there are 61 ground truth BFRSs covered by trajectory dataset 1. Out of these, 37 BFRSs are correctly detected and 24 BFRSs remain undetected. Therefore, the numbers for correctly detected BFRSs, falsely detected BFRSs, and undetected BFRSs are 37, 0, and 24 respectively. The *Precision*, *Recall*, and *F1-score* are computed as 1.0000, 0.6066, and 0.7551, respectively. In trajectory dataset 2, there are 39 BFRSs distributed along the trajectory. Out of these, 34 are detected, one is falsely detected, and none are undetected. The *Precision*, *Recall*, and *F1-score* for dataset 2 are computed as 0.9706, 0.8462, and 0.9041, respectively. On the other hand, for dataset 3, the *Precision*, *Recall*, and *F1-score* are computed as 1.0000, 0.7931, and 0.8846, respectively. The proposed method performs well in the experiment, which is consistent with the result shown in Figure 12. Detailed statistics for other trajectory datasets are also conducted, and different BFRSs are detected from the trajectory. The number of detected BFRSs ranges from 4 to 62, while the number of correctly detected BFRSs ranges from 4 to 57, and the number of undetected BFRSs ranges from 4 to 24. By combining these results with the information in Figure 13 and Table 2, it can be observed that not all ground truth BFRSs are completely detected by a single trajectory dataset. However, an increasing number of BFRSs are being detected from crowd-sourced trajectory datasets, which allows for the sensing and representation of road surface conditions. Across the trajectory datasets, the *F-Scores* range from 0.5333 to 0.9123, with most scores indicating a strong balance between *Precision* and *Recall*. This suggests that the method, despite its variability in *Precision* and *Recall*, maintains a robust overall performance in detecting BFRSs. In a cumulative analysis, 62 BFRSs are correctly detected out of 71 detected results, while 9 BFRSs are falsely detected and 3 remain undetected out of the 65 ground truth BFRSs, as depicted by red rectangles in Figure 13. Hence, a field survey is conducted for the undetected BFRSs in the research area, as depicted in Figure 14. It can be observed that these BFRSs are located at the centerline of the road, covered by nearly flat asphalt surfaces. Although the size is large and there are bump features at the rim of the circular object, it is difficult to capture acceleration changes while driving, necessitating more crowd-sourced trajectories. From these results, the sum row highlights an aggregated *Precision* of 0.8732 and *Recall* of 0.9538, leading to an overall *F-Score* of 0.9118. 

In the above experiments, the analysis demonstrates the potential of using crowd-sourced trajectories for sensing road surface conditions. Although the detection of BFRSs may not be fully achieved from a single trajectory, the results can be refined, and more BFRSs can be detected from the crowd-sourced dataset. In addition, the proposed method can handle trajectories from different data sources, and is independent of any specific smartphone or BFRS. The high precision and recall rates suggest that the method is accurate, reliable, and capable of making significant contributions to urban road maintenance and planning.

## 5. Conclusions and Future Work

This article emphasizes the importance of road surface condition sensing in urban road maintenance and planning. It highlights the increasing relevance of smartphones as a source of crowd-sourced data for road surface information, such as BFRSs. The research presented in this article focuses on analyzing crowd-sourced trajectories from smartphones. It involves conducting spatial transformations of recorded acceleration data, computing heading angles, and extracting vehicle movement features based on multiple sensor recordings. Wavelet scattering transformations are then applied to encode BFRSs. The article also discusses the design of a neural network for BFRS detection and proposes a two-stage clustering method for representing and integrating BFRSs from crowd-sourced trajectories. The effectiveness of the proposed method based on the DCF is demonstrated through experiments using crowd-sourced trajectories from smartphones with multiple sensors. Additionally, comparisons between the proposed method and other BFRS detection methods are conducted, revealing that the BFRS detection results obtained with the proposed method outperform those of other methods. In the research area comprising 32 trajectories, 62 out of 65 ground truth BFRSs are correctly detected, with *F-Scores* ranging from 0.5333 to 0.9123. Although BFRSs may not be totally detected in just a single trajectory, the result can be refined with crowd-sourced datasets. Consequently, the overall *F-Score* improves to 0.9118, with an aggregated *Precision* of 0.8732 and *Recall* of 0.9538, further showcasing the capabilities of this approach. This methodology has significant implications for the maintenance and planning of urban roads, contributing to safer traffic navigation and improved city planning.

Future research should focus on integrating non-homogeneous datasets with multiple sampling frequencies and qualities to enhance the robustness of the method. Another aspect that needs further exploration is urban road surface condition sensing using multi-modal sensor datasets, such as video, acceleration, and LiDAR datasets, which can detect both geometric shapes and GPS locations.

## Figures and Tables

**Table 1:** The statistics of the research area.

Dataset	*A* (m^2^)	*L_A_* (m)	*N_traj_*	*L_traj_* (m)	*R_GPS_*	*R_acc_*_(3-axis)_	*R_ori_*_(3-axis)_
*Ds*	290,972	2410	32	33,559	3898	389,780	389,780

**Table 2:** The statistics of road surface sensing results from crowd-sourced datasets.

Dataset ID	Ground Truth BFRSs	Detected BFRSs	TT BFRSs	FT BFRSs	TF BFRSs	*Precision*	*Recall*	*F-Score*
1	61	37	37	0	24	1.0000	0.6066	0.7551
2	39	34	33	1	6	0.9706	0.8462	0.9041
3	29	23	23	0	6	1.0000	0.7931	0.8846
4	48	34	33	1	15	0.9706	0.6875	0.8049
5	30	27	26	1	4	0.9630	0.8667	0.9123
6	61	50	48	2	13	0.9600	0.7869	0.8649
7	43	29	28	1	15	0.9655	0.6512	0.7778
8	31	14	14	0	17	1.0000	0.4516	0.6222
9	19	10	10	0	9	1.0000	0.5263	0.6897
10	33	28	27	1	6	0.9643	0.8182	0.8852
11	23	20	19	1	4	0.9500	0.8261	0.8837
12	15	10	10	0	5	1.0000	0.6667	0.8000
13	15	9	8	1	7	0.8889	0.5333	0.6667
14	61	41	40	1	21	0.9756	0.6557	0.7843
15	65	62	57	5	8	0.9194	0.8769	0.8976
16	32	17	16	1	16	0.9412	0.5000	0.6531
17	32	22	20	2	12	0.9091	0.6250	0.7407
18	32	22	20	2	12	0.9091	0.6250	0.7407
19	29	23	21	2	8	0.9130	0.7241	0.8077
20	32	18	17	1	15	0.9444	0.5313	0.6800
21	21	13	12	1	9	0.9231	0.5714	0.7059
22	11	5	5	0	6	1.0000	0.4545	0.6250
23	32	19	18	1	14	0.9474	0.5625	0.7059
24	31	24	22	2	9	0.9167	0.7097	0.8000
25	31	21	19	2	12	0.9048	0.6129	0.7308
26	11	4	4	0	7	1.0000	0.3636	0.5333
27	31	18	18	0	13	1.0000	0.5806	0.7347
28	32	19	18	1	14	0.9474	0.5625	0.7059
29	32	22	21	1	11	0.9545	0.6563	0.7778
30	31	23	22	1	9	0.9565	0.7097	0.8148
31	21	10	9	1	12	0.9000	0.4286	0.5806
32	31	23	22	1	9	0.9565	0.7097	0.8148
**sum**	65	71	62	9	3	0.8732	0.9538	0.9118

## Data Availability

Publicly available datasets were analyzed in this research, and they can be found at: https://doi.org/10.6084/m9.figshare.25904482. The street map is available from OpenStreetMap accessed on 20 May 2024.
